# Current Understanding of the Pathophysiology of Myocardial Fibrosis and Its Quantitative Assessment in Heart Failure

**DOI:** 10.3389/fphys.2017.00238

**Published:** 2017-04-24

**Authors:** Tong Liu, Deli Song, Jianzeng Dong, Pinghui Zhu, Jie Liu, Wei Liu, Xiaohai Ma, Lei Zhao, Shukuan Ling

**Affiliations:** ^1^Department of Cardiology, Capital Medical University, Beijing AnZhen HospitalBeijing, China; ^2^Department of Cardiology, Beijing Changping HospitalBeijing, China; ^3^Department of Vascular Surgery, Chinese PLA General HospitalBeijing, China; ^4^Vascular Surgery Research Laboratories, Division of Vascular and Endovascular Surgery, Brigham and Women's Hospital, Harvard Medical SchoolBoston, MA, USA; ^5^Department of Radiology, Beijing Anzhen Hospital, Capital Medical UniversityBeijing, China; ^6^State Key Lab of Space Medicine Fundamentals and Application, China Astronaut Research and Training CenterBeijing, China

**Keywords:** mycardial fibrosis, heart failure, late gadolinium enhancement, micro RNAs (miRNAs), biomarkers, extracelluar matrix

## Abstract

Myocardial fibrosis is an important part of cardiac remodeling that leads to heart failure and death. Myocardial fibrosis results from increased myofibroblast activity and excessive extracellular matrix deposition. Various cells and molecules are involved in this process, providing targets for potential drug therapies. Currently, the main detection methods of myocardial fibrosis rely on serum markers, cardiac magnetic resonance imaging, and endomyocardial biopsy. This review summarizes our current knowledge regarding the pathophysiology, quantitative assessment, and novel therapeutic strategies of myocardial fibrosis.

## Introduction

Heart failure (HF) is a malignant and fatal disease, causing medical, and financial burdens worldwide (Yancy et al., 2013). A key mechanism of HF is cardiac remodeling, which includes two aspects: cardiomyocyte injury and myocardial fibrosis (Heusch et al., 2014). Cardiomyocyte injury presents as cardiomyocyte hypertrophy, necrosis, and apoptosis. Numerous researchers focused on this area of study over the past decades. However, the efficacy of the available treatment options for patients with HF is still undesirable. Recently, many investigators shifted their focus to myocardial fibrosis to explore more effective treatments. The present review summarizes the current literature on myocardial fibrosis and focuses on its pathogenesis, clinical manifestation, detection methods, prognosis, and strategies to manage myocardial fibrosis in HF.

## Epidemiology and risk factors

Heart failure is becoming a growing epidemic that poses significant clinical and economical challenges. The incidence of HF is about 1–2% in developed countries and increased to 10% among people over 70 years of age. The lifetime risk of HF is significantly higher in men than in women, but there is no difference in prognosis. Heart failure is a fatal disease with only 35% of patients surviving 5 years after the first diagnosis (Bleumink et al., 2004). According to the newest European Society of Cardiology guidelines regarding HF, the categories include HF with reduced ejection fraction (HFrEF; left ventricular ejection fraction [LVEF] < 40%), HF with preserved ejection fraction (HFpEF; LVEF ≥50%), and HF with mid-range ejection fraction (HFmrEF; LVEF in the range of 40–49%). These patients undergo myocardial fibrosis in all types of HF (Ponikowski et al., 2016). As demonstrated by the increasing number of acute coronary events, ischemic heart disease has been an important cause of HF in the past half-century. Despite the prevalence of ischemic heart disease, other diseases can contribute to alterations in the structure and function of the heart and ultimately cause the occurrence of HF. These diseases include hypertension, dilated cardiomyopathy (DCM), hypertrophic cardiomyopathy (HCM), and valve heart disease. Age-associated myocardial fibrosis is also partially responsible for HF, especially in patients with HFpEF (Horn and Trafford, 2016).

## Pathogenesis

There are two types of myocardial fibrosis: replacement and interstitial fibrosis (Ambale-Venkatesh and Lima, 2015). The former usually occurs as a result of myocyte necrosis after myocardial infarction. Other conditions associated with replacement fibrosis include hypertrophic cardiomyopathy, sarcoidosis, myocarditis, chronic renal insufficiency, and toxic cardiomyopathies (Table 1; Burt et al., 2014). The latter is diffuse, and its subtypes include reactive and infiltrative interstitial fibrosis. Reactive fibrosis is present in many types of diseases, including aging and hypertension. Infiltrative fibrosis is relatively rare and is caused by progressive deposition of insoluble proteins (amyloidosis) or glycosphingolipids (Anderson-Fabry disease) in the interstitial space (Hashimura et al., 2017). Eventually, both interstitial and infiltrative fibrosis can lead to cardiomyocyte apoptosis and replacement fibrosis (Hashimura et al., 2017). Regardless of its type, myocardial fibrosis is a complicated process resulting in the accumulation of the extracellular matrix (ECM). Therefore, it is important to understand the ECM's physiological structure and its pathological changes during HF.

**Table 1:** **Cardiac diseases that cause myocardial fibrosis**.

**The models of myocardial fibrosis**	**Cardiac diseases**
Replacement fibrosis	Myocardial infarction, sarcoidosis, myocarditis, toxic cardimyopathies, chronic renal insufficiency
Reactive interstitial fibrosis	Hypertension, diabetes, non-ischemic dilated cardiomyopathy, hypertrophic cardiomyopathy, sarcoidosis, chronic renal insufficiency
Infiltrative interstitial fibrosis	Amyloidosis, Anderson-Fabry disease

### ECM synthesis

The myocardial ECM contains various proteins and signaling molecules that maintain a dynamic balance and support the integrity of the cardiac structure (Bonnans et al., 2014). The ECM formulates the scaffold that surrounds cardiomyocytes and intramural coronary vasculature to inhibit myofibril slippage and support the cardiac tissue's mechanical function (Weber et al., 2013; Bonnans et al., 2014). Moreover, it provides a link between intracellular cytoskeletal proteins and intercellular proteins, which allows the heart to transmit biochemical signals through mechanosensation (Weber et al., 2013). These links play a critical role in the activation and differentiation of myofibroblasts. In general, ECM synthesis contributes to myocardial fibrosis and eventually results in structural changes of the heart tissue and cardiac insufficiency.

#### Cellular origins of ECM-myofibroblasts

**Figure 1:**
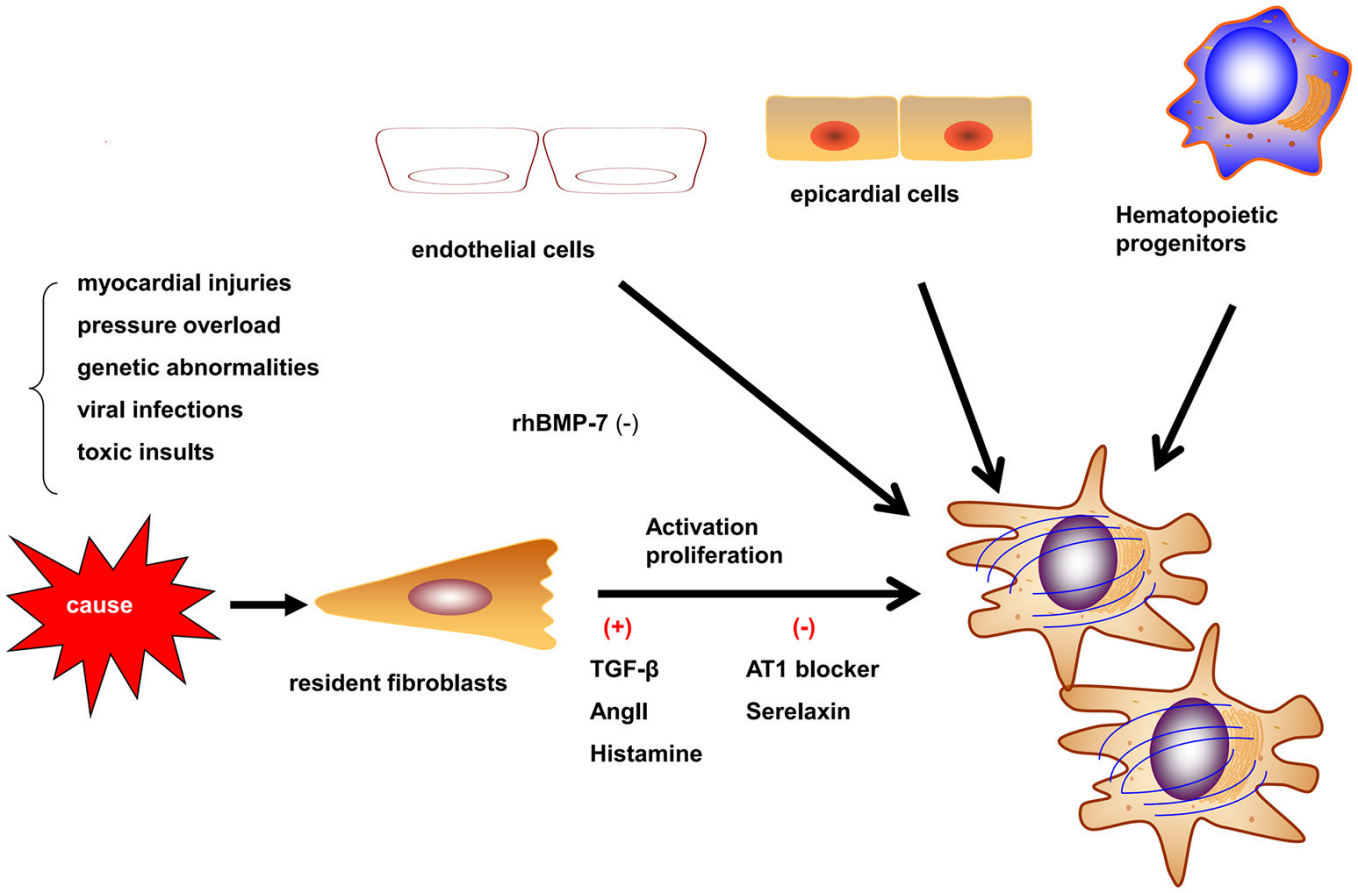
**Origins and activation of myofibroblasts**. Myofibroblasts can be activated in different situations such as myocardial injury, pressure overload, and genetic abnormalities. Myofibroblasts may be derived from resident fibroblasts, bone marrow-derived fibroblasts, epithelial-to-mesenchymal transition, and endothelial-to-mesenchymal transition (EndMT). The contribution of different origins may be variable in different etiologies.

Myofibroblasts are the most prominent cells involved in myocardial fibrosis. Myofibroblasts are not present until their precursor cells are activated by different causes such as myocardial injuries, pressure overload, genetic abnormalities, viralinfections, and toxic insults. These causes activate myofibroblasts by mechanical conductor signals and signaling molecules, including TGF-β1, endothelin-1, fibroblast growth factor, and cytokines (e.g., IL-1, IL-6, and TNF-α; Schroer and Merryman, 2015). The myofibroblasts may be derived from resident fibroblasts, bone marrow-derived fibroblasts, epithelial-to-mesenchymal transition (EMT) and endothelial-to-mesenchymal transition (EndMT; Figure 1; Kong et al., 2014). The different etiologies of fibrosis may result from the fibroblasts of different origins. In the myocardial infarct model, most myofibroblasts are derived from hematopoietic fibroblast progenitors (Mollmann et al., 2006). Zeisberg et al. reported that EndMT may lead to cardiac fibrosis in mouse models of pressure overload and chronic allograft rejection. Furthermore, TGF-β1-induced EndMT in adult coronary endothelial cells was reported to lead to cardiac fibrosis. In contrast, the recombinant human BMP-7 (rhBMP-7) inhibits EndMT and cardiac fibrosis in mouse models by preserving the endothelial phenotype (Zeisberg et al., 2007). However, Moore-Morris et al. drew a different conclusion by lineage tracing, whereby, following a pressure overload, the implicated fibroblasts were derived from the resident epicardial- and endocardial-derived fibroblasts and endocardium during embryonic development, but not from the EndMT, epicardial EMT, or the accumulation of hematopoietic fibroblast progenitors (Moore-Morris et al., 2014). Therefore, understanding the derivation, proliferation, and activation of fibroblasts is required to develop anti-fibrotic therapies. In particular, discovering reasonable biomarkers of myofibroblasts should be the focus of future studies.

#### Collagen deposition

Activated myofibroblasts can redundantly express ECM-related genes to synthesize several types of collagens, including collagen I and collagen III. Following various processing steps on pro-collagen, mature collagen can be deposited within the extracellular space. Furthermore, collagen must be cross-linked by lysyl oxidase to generate effective fibers that can resist proteinase degradation. During fibrosis, collagen I, providing rigidity, increases more apparently than collagen III, providing elasticity, which contributes to an increased wall tension (Segura et al., 2014). Various cells (e.g., inflammatory cells) and mediators (such as cytokines, growth factors, and hormones) are involved in collagen turnover. Some investigators demonstrated that the ECM itself can also contribute to added ECM production (Bonnans et al., 2014). For instance, myofibroblasts release signaling factors such as TGF-β1, which promote further myofibroblast differentiation and ECM remodeling. Some reports proposed that aging is an independent risk factor that can induce cardiac remodeling, although, the main reason may be collagen deposition, which increases with age and commonly reduces the heart's diastolic function (Horn and Trafford, 2016).

### ECM degradation

Matrix metalloproteinases (MMPs) play pivotal roles in the development of fibrosis by controlling ECM degradation. Diverging from previous viewpoints, recent studies showed that MMPs are not only involved in ECM degradation, but are also implicated in the progression to HF (Spinale et al., 2013). The family of MMPs include collagenases such as MMP-1, MMP-13, and MMP-8; gelatinases such as MMP-2 and MMP-9, among others. In animal models, different causes of HF may induce different MMPs (Spinale et al., 2013). For example, MMP-2 and MMP-14 levels robustly increase in LV pressure overload models and myocardial biopsies of aortic stenosis (AS), whereas the levels of MMP-14, MMP-7, MMP-8, and MMP-9 increase in end-stage dilated cardiomyopathy. The level of MMP-2 is higher than that of MMP-9 in early-stage HF, but the level of MMP-9 exceeds that of MMP-2 during end-stage HF. Many researchers studied the role of MMP-9 in cardiac fibrosis in senescent hearts without any other cardiac injury, which is associated with the higher possibility of HFpEF in aging individuals. Munch et al. found that serum MMP-9 is a useful biomarker for myocardial fibrosis and sudden cardiac death (SCD) in female patients with HCM, whereas MMP-3 is associated with a higher rate of cardiac events independent of factors such as fibrosis or sex (Munch et al., 2016). MMP-14, the membrane-type MMP, is involved in many different pathogeneses of LV remodeling. MMP-14 is primarily involved in the accelerated proteolysis of ECM molecules or activating MMP-2 to degrade the ECM. Alternatively, MMP-14 can also promote ECM synthesis by increasing the function of TGF (Spinale et al., 2013). The tissue inhibitor of metalloproteinases (TIMP) family, which can inhibit the activity of MMPs, is composed of four members: TIMP-1, -2, -3, and -4; each with unique binding specificities for particular MMPs. TIMPs are also involved in myocardial fibrosis and HF (Bonnans et al., 2014).

In particular, TIMP-4 may lead to the development of cardiac fibrosis by inhibiting MMP-9 activity. Collectively, the relative levels of MMPs and TIMPs determine the overall rate of ECM degradation. Pressure or volume overload, myocardial infraction, myocarditis, and other factors may cause the imbalance of different MMPs and TIMPs, leading to the development of myocardial fibrosis and HF.

### The roles of inflammatory cells in myocardial fibrosis

#### Macrophages

**Figure 2:**
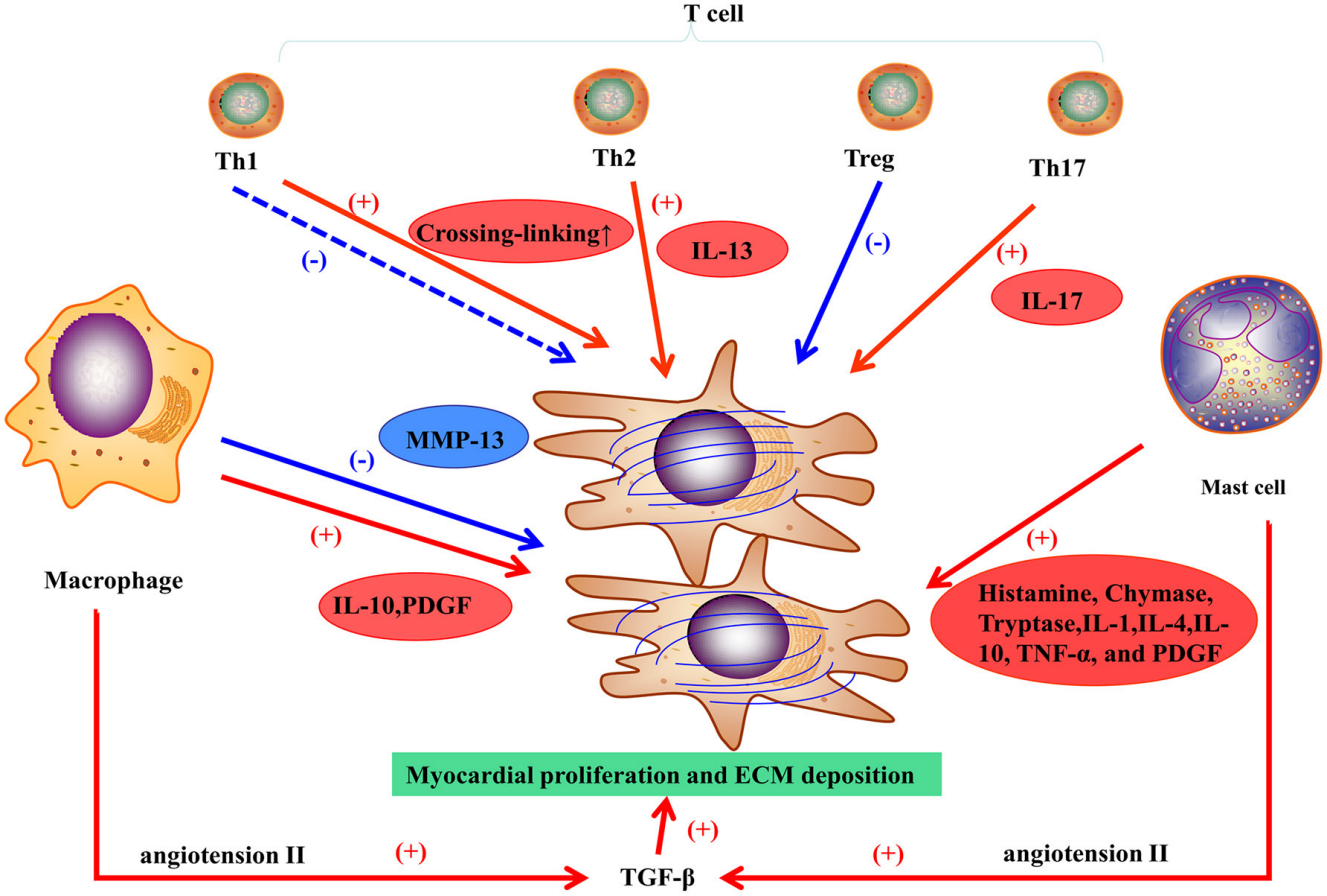
**Inflammatory response in myocardial fibrosis**.

Macrophages play a vital role in pro-fibrotic response and regulation of fibrosis. In the healthy heart, monocytes, the progenitor cells of macrophages, are maintained in a steady state, but can differentiate into macrophages during cardiac injury regardless of the etiology. Although, macrophages may differentiate *in situ* from monocytes, they derive mostly from the recruitment of monocytes from the circulation, and inhibiting macrophages infiltration may prevent the development of fibrosis (Falkenham et al., 2015). For example, the infarcted myocardium can release endogenous signals, referring to danger-associated molecular patterns (DAMPS), to promote local monocyte proliferation and to mobilize bone marrow-derived monocytes. Complement effector and C-C motif chemokine 2 (CCL-2) have also been involved in monocyte recruitment (Ruparelia et al., 2017). Cardiac remodeling is regulated by different subsets of macrophages expressing heterogeneous cell surface markers. M1 and M2 are the two subpopulations of macrophages classified *in vitro* (Murray and Wynn, 2011). For example, in an experimental trial, M1 macrophages were recruited to the infarcted zone during the inflammatory stage of myocardial stenosis, then exhibited a proteolytic activity and secreted pro-inflammatory mediators (including IL-1, TNF, and ROS). M2 macrophages, following pro-inflammatory cells or transiting from M1 macrophages, exhibit an anti-inflammatory response and have a crucial function in wounding healing and fibrosis. M2 macrophages can release pro-fibrotic mediators (Figure 2; such as IL-10, TGF-β, and PDGF) and chemokines that recruit fibroblasts (Hulsmans et al., 2016). Otherwise, M2 macrophages can inhibit fibrosis by phagocytosing apoptotic myofibroblasts and regulating the balance of MMPs and TIMPs (Hulsmans et al., 2016). A study on hepatic fibrosis showed that macrophages could express high levels of MMP-13 and suppress fibroblast activation to resolve fibrosis (Fallowfield et al., 2007). Only applying M1/M2 classification to humans is too simple to represent the heterogeneity of macrophages (Murray and Wynn, 2011). Therefore, different subsets of macrophages with distinct properties and their communication with other cells have been further investigated.

#### Mast cells

Mast cells participate in myocardial fibrosis primarily via pro-fibrotic and inflammatory functions (Levick et al., 2011). Increasing density of mast cells has been showed in ischemic cardiomyopathy (Engels et al., 1995) and hypertension (Panizo et al., 1995) in animal models. In DCM patients with end-stage HF, mast cell density is correlated with the collagen fraction that represents myocardial fibrosis in tissue sampling (Batlle et al., 2007). Levick and co-workers found that mast cell stabilization prevented the left ventricular fibrosis in spontaneously in a hypertensive rat model (Levick et al., 2009). Generally, mast cells may play a vital role in myocardial fibrosis in HF, but definite mechanisms have not been clarified. Mast cells can release many substances by degranulation such as histamine, tryptase, and chymase to mediate fibrosis (Figure 2).

Histamine stimulates fibroblast proliferation in pulmonary fibrosis (Jordana et al., 1988). Additionally, activating histamine H2 receptors in cardiomyocytes contributes to the increase in the production of cyclic adenosine monophosphate (cAMP) in the failing heart. Excessive cAMP increases oxygen consumption and worsens the heart function. Blocking H2 receptor improves HF symptoms and ventricular remodeling (Kim et al., 2006). A prospective study also showed that an inhibitor of H2 receptors can reduce HF incidence and age-related left heart morphology change (Leary et al., 2016). Therefore, histamine may have an important function in cardiac remodeling, and inhibiting H2 receptor may be an important target to improve HF prognosis under the current pharmacotherapy. Chymase, a mast cell specific protease, enhances fibrogenic activity by increasing the abundance of angiotensin II and TGF-β in a manner that cannot be blocked by ACE inhibitors. A study showed that pretreatment with TGF-β1 neutralizing antibody suppressed chymase-induced collagen production. However, the blockade of angiotensin II receptor had no effect on chymase-induced production of TGF-β1 and pro-fibrotic action (Zhao et al., 2008). According to this study, chymase may promote myocardial fibrosis via the TGF-β1/Smad pathway rather than angiotensin II. Nevertheless, chymase influences MMP activity to modulate ECM synthesis. Oyamada et al. demonstrated that a chymase inhibitor reduced the infarction size and MMP-9 activation and attenuated fibrosis after acute myocardial ischemia/reperfusion in a porcine model (Oyamada et al., 2011). Tryptase, another product secreted by mast cells, can activate MMP-1 and MMP-3 in skin mast cells (Levick et al., 2011). However, a recent study noted that systemic levels of mast cell tryptase was lower in LV systolic dysfunction, LV dilatation, or clinical CHF, which may result from the local consumption of tryptase (Upadhya et al., 2004). Therefore, local mast cell contribution to myocardial fibrosis may be undetectable through systemic sampling. In addition, a majority of cytokines and growth factors can be released by mast cells, including IL-1,-4,-10, TNF-α, and PDGF. However, these products can also be secreted by other inflammatory cells. Thus, understanding the derivation and function of these molecules may help us identifying the mechanism underlying the role of inflammatory cells in fibrosis.

#### Lymphocytes

Infiltration of lymphocytes, mainly T cells, is associated with the progression of heart failure. Nevers et al. showed that T cells from non-ischemic HF patients or from mice with heart failure induced by transverse aortic constriction had higher affinity and enhanced adhesion for the activated vascular endothelium, compared with those from healthy subjects or sham mice (Nevers et al., 2015). The recruitment and infiltration may contribute to pathological cardiac remodeling in HF. In rodent models of experimental autoimmune myocarditis, T cells are activated and a peak stage of inflammation and necrosis occurs around 21 days. Inflammatory cells infiltration declines and is replaced with fibrosis after 21 days, eventually leading to dilated cardiomyopathy and heart failure (Watanabe et al., 2011). Hofmann et al. demonstrated that CD4+ T cells are activated in mice with MI, apparently decreasing mortality, but possibly responsible for further wound healing of the myocardium (Hofmann et al., 2012).

In general, T cells can differentiate into four subpopulations, T helper cells (including Th1 and Th2 cells), regulatory T cells (Tregs), and Th17 cells (Wei, 2011). Th1 cells are involved in acute inflammation response and exert anti-fibrotic activities by secreting cytokines. A recent study showed that Th1 cells can also mediate left ventricular collagen cross-linking, leading to diastolic dysfunction in mice (Yu et al., 2010). Th2 cells participate in chronic remodeling response and contribute to fibroblast activation, proliferation, and matrix accumulation. Th2 cells release IL-4 and IL-13. The latter induces fibrosis by stimulating collagen production (Wei, 2011). Tregs can attenuate myocardial fibrosis in an animal model of MI (Tang et al., 2012) and hypertension (Kvakan et al., 2009); the main mechanisms involve the inhibition of pro-inflammatory cytokines and the protection of cardiomyocytes from injury. Th17 cells affect myocardial fibrosis through production of IL-17, which promotes collagen production and may modulate cytokine, collagen, or MMP/TIMP mRNA stability, thereby contributing to myocardial fibrosis (Figure 2; Wei, 2011).

### Other molecules regulating myocardial fibrosis

Inflammatory cytokines, reactive oxygen species, TGF-β, and the renin-angiotensin-aldosterone system all regulate the process of myocardial fibrosis (Kong et al., 2014). Recent studies described some emerging molecules such as cardiotrophin-1, nicotinamide adenine dinucleotide phosphate oxidase, and various matricellular proteins that are also involved in myofibroblast activation, collagen turnover, and collagen cross-linking (Heymans et al., 2015). These small molecules are also under investigation as diagnostic and therapeutic targets.

Matricellular proteins do not exist in the normal ECM, but they are upregulated during myocardial injury and stress. Matricellular proteins can transduce signals between matrix proteins and cells, including thrombospondins, and osteopontins (Kong et al., 2014).

TGF-β leads to myocardial fibrosis by activating the differentiation of fibroblasts into myofibroblasts and accelerating ECM deposition. Moreover, it can suppress the degradation of the ECM by inhibiting MMPs and enhancing the activity of TIMPs. There are threeTGF-βisoforms: TGF-β1, TGF-β2, and TGF-β3 (Segura et al., 2014).

TGF-β1 is the main isoform and is involved in cardiac fibrosis. In normal circumstances, TGF-β is in its static form and becomes active during cardiac injury. Small quantities of TGF-β can contribute to an extensive intracellular response. TGF-β binds and activates the TGF-β receptor TβR-II, activating the Smad pathway through the intracellular receptor TβR-I. Therefore, the Smad3 pathway is essential for TGF-β1-induced myocardial fibrosis (Segura et al., 2014). In addition, TGF-β1 can mediate the functions of other important molecules and neurohormones such as angiotensin (Ang) II. Beyond its action on macrophages, Ang II can upregulate TGF-β1 activity in myofibroblasts via the AT_1_ receptor (Figure 2; Weber et al., 1989). Finally, genes coding for ECM proteins (e.g., collagen I and III proteins) become more active, which results in the accumulation of fibrous tissues. Cardiac fibrosis induced by TGF-β1 is associated to cardiomyopathy, valve thickening, valve dysfunction, and electrophysiological abnormalities. In animal models, inhibition of TGF-β1 can prevents myocardial fibrosis to attenuate the progression of diastolic dysfunction (Kuwahara et al., 2002).

Altogether, it appears that ECM synthesis, post-translational modifications, and ECM degradation are highly regulated processes, and even slight changes to these processes may have drastic effects on the cardiac structure and function. Myocardial fibrosis in HF, which can be the result of an abnormality in these pathways, results in systolic dysfunction, diastolic stiffness, and aberrant ion conduction.

## Clinical diagnostic methods and prognostic significance

Myocardial fibrosis is a common characteristic of HF, but the pattern of myocardial fibrosis depends on the etiology of HF. Myocardial fibrosis manifests QRS prolongation (Loring et al., 2013), frequent ventricular premature beats, and ventricular tachycardia (VT) on electrocardiogram. The ultrasonic cardiogram, a basic instrument used to evaluate cardiac structure and function, can detect increased left ventricular end diastolic diameter, decreased ejection fractions, systolic dyssynchrony, and elevated filling pressures. Diffuse myocardial fibrosis may lead to impaired movement of the entire ventricular wall. However, these techniques have no specificity for the detection of fibrosis. Therefore, new diagnostic methods have been developed to detect fibrosis and evaluate its extent more effectively. Presently, the extent of myocardial fibrosis can be evaluated by using different methods, including endomyocardial biopsy, magnetic resonance imaging (MRI), or with serum markers of collagen turnover (Aoki et al., 2011).

We will discuss both the pros and cons of each detection method and the significance to indicate prognosis of HF. First, we should discuss the current standard for evaluating HF prognosis. In current studies, the main causes of death in patients with HF are pump function failure or SCD (Ahmad et al., 2014). Myocardial fibrosis is involved in the progression of LV dilatation and deterioration of the cardiac muscle, leading to pump function failure. Pump function failure, cardiomyocyte separation, and fibrosis itself may induce fatal arrhythmias. Therefore, when evaluating an indicator for its prognostic significance, we should estimate it in two aspects: pump function failure and SCD.

### Serum markers

#### Galectin-3 and ST-2

Myocardial fibrosis is an ongoing remodeling process that requires the discovery of effective and specific biomarkers in order to diagnose and provide therapeutic interventions. In 2013, the ACCF/AHA Heart Failure Guidelines recommended the use of two myocardial fibrosis markers, galectin-3 and soluble ST2, for risk stratification with Class IIb recommendations (Yancy et al., 2013).

Galectin-3 (Gal-3) is secreted by inflammatory cells and fibroblasts, and can be degraded by MMPs. Some animal experiments demonstrated that Gal-3 can accelerate myocardial fibrosis in cardiac disease. Moreover, circulating levels of Gal-3 are associated with the degree of myocardial fibrosis and can predict the re-hospitalization and all-cause mortality of HF. In addition, Gal-3 is an excellent marker for the detection of earlier cardiac remodeling without HF (Ho et al., 2012).

ST2, a member of the interleukin-1 receptor family, exists in both a transmembrane and a soluble form. The transmembrane form is called ST2L. Interleukin-33 (IL-33) plays a role in suppressing myocardial fibrosis and cardiomyocyte hypertrophy via binding to ST2L, whereas excessive soluble ST2 can attenuate the function of IL-33, leading to myocardial fibrosis and ventricular dysfunction. Many studies indicated that ST2 is associated with all-cause mortality and cardiovascular mortality (Passino et al., 2015).

In recent years, there have been many studies on the functions of ST2 and Gal-3 as clinical diagnosis and prognosis markers. Bayes-Genis et al. reported that adding an ST2 evaluation can help clinicians to classify patients with HF (Bayes-Genis et al., 2014). However, in another study, the incorporation of ST2 and Gal-3 to clinical evaluations could not significantly predict pump failure of patients with HF, whereas it could indicate the SCD risk (Ahmad et al., 2014).

#### MicroRNAs

ECM deposition requires an increase in the expression of ECM-related proteins such as proteins involved in collagen synthesis. MicroRNAs (miRNAs) are a cluster of short RNAs that can regulate these processes by promoting either the degradation or the translational repression of their target mRNAs, while playing a vital role in myocardial fibrosis (van Rooij and Olson, 2012; Thum, 2014). Many researchers investigated the role and mechanism of miRNAs in HF. We first briefly introduce currently studied miRNAs. Circulating levels of miR-21 are related to the grade of myocardial fibrosis. Villar et al. showed that miR-21 levels in patients with AS are prominently higher than those in control patients. Moreover, miR-21 is associated with the echocardiographic mean transvalvular gradients (Villar et al., 2013). At the cellular level, miR-21 can activate fibroblasts and induce EMT and EndMT. In addition, it can affect the process of fibrosis at the molecular level by targeting the endogenous mitogen-activated protein kinase inhibitor sprouty-1 (SPRY1), matrix metalloproteinase-2, and TGF-β RIII. Animal studies showed that miR-21 enhances the ERK-MAPK signaling pathway in mouse fibroblasts and heart samples by inhibiting SPRY1 expression, leading to fibroblast survival. Additionally, the anti-miR-21 antagomir can repress the expression of genes encoding collagen and ECM proteins. miR-29 has also been investigated in myocardial fibrosis. miR-29 represses the expression of ECM genes, such as those encoding collagens, elastins, or fibrillins. However, the repressive function was reduced due to miR-29 decrease during HF. Additionally, miR-29b can directly inhibit proteins involved in building the ECM such as collagens, MMPs, leukemia inhibitory factors, and insulin-like growth factor I. Roncarati et al. reported that miR-29a is associated with both cardiomyocyte hypertrophy and myocardial fibrosis when patients with HCM and control subjects were comparatively evaluated, thus identifying miR-29a as a potential biomarker to assess myocardial remodeling during HCM (Roncarati et al., 2014). miR-208, encoded by the introns of myosin, is associated with cardiac remodeling, whereby knocking down miR-208a can alleviate fibrosis and cardiac hypertrophy in a mouse model (van Rooij et al., 2007). In summary, some miRNAs are upregulated in HF, whereas some are downregulated. Therefore, there are many studies about the cluster of miRNAs involved in myocardial fibrosis.

#### Pro-collagen type I (PICP) and amino-terminal pro-peptide of pro-collagen type III (PIIINP)

Besides other serum markers, current molecules proven to associate with myocardial fibrosis (as measured by histological parameters and collagen volume fraction) are the carboxy-terminal pro-peptide of pro-collagen type I (PICP) and the amino-terminal pro-peptide of pro-collagen type III (PIIINP; reviewed by Prockop and Kivirikko, 1995). PICP originates from the conversion of pro-collagen type I into collagen type I, and similarly, PIIINP originates from the conversion of pro-collagen type III into collagen type III. Serum PICP levels correlate with total collagen volume fraction (CVF) in HF patients with hypertensive heart disease, but not in HF patients with ischemic heart disease or idiopathic dilated cardiomyopathy (DCM). Moreover, serum PIIINP levels correlate with the myocardial collagen type III volume fraction (CIIIVF) in HF patients with ischemic heart disease or idiopathic DCM (Lopez et al., 2015). However, the greatest disadvantage of the two biomarkers is their low specificity. Organ fibrosis such as hepatic fibrosis and pulmonary fibrosis could lead to the apparent increase in these markers. In another study, Lopez-Andre et al. found that increased levels of Gal-3 and PIIINP, and a decreased level of MMP-1, are associated with death or HF hospitalization, while neither marker could predict a response to cardiac resynchronization therapy (CRT). Here, CRT did not influence serum concentrations of ECM markers, which indicated no effect of alleviating myocardial fibrosis by CRT (Lopez-Andres et al., 2012).

Therefore, finding better biomarkers of myocardial fibrosis requires a deeper understanding of the molecular mechanisms underlying the development of fibrosis. These molecules and each part of the pathway contributing to fibrosis can be targeted as biomarkers for anti-fibrotic therapy or to predict a prognosis for patients with HF.

### Cardiac magnetic resonance (CMR) imaging

#### Late gadolinium enhancement (LGE)

Myocardial fibrosis causes the enlargement of the extracellular space because of ECM deposition and excessive retention of gadolinium leads to enhancement in CMR (Karamitsos et al., 2009). Different etiologies causing myocardial fibrosis can show different patterns of LGE (Karamitsos et al., 2009; Doltra et al., 2013). Therefore, MRIs can help us to identify the cause of HF, especially for cardiomyopathies.

#### LGE patterns of ischemic cardiomyopathy

Most patients with DCM will show no LGE because diffuse myocardial fibrosis cannot be observed with an LGE-MRI, but some others may present with a mid-wall hyper-enhancement and fewer cases show patchy or diffuse striated LGE. The distribution of LGE is unrelated to a particular coronary arterial territory (Zacharski et al., 2015). For DCM patients with ventricular arrhythmias, LGE-CMR can help determining the origin of the ectopic pacemaker and guide the establishment of a therapeutic schedule. The LGE pattern in the setting of myocarditis presents a characteristic distribution (Lagan et al., 2017). The subepicardium is usually affected with varying degrees of progression toward the mid-myocardial wall and typical sparing of the subendocardium, and the lateral and inferolateral walls are frequently involved (Andre et al., 2016). The focal areas of hyper-enhancement become diffuse over a period of days to weeks and then decrease during healing. However, some patients cannot recover and often develop DCM, which usually presents as a mid-wall hyper-enhancement. Asymmetrical thickness of the interventricular septum and LV outflow track obstruction are the main characteristics of HCM. The hyper-enhancement of HCM usually affects the mid-wall, especially at LV–RV junctions. In burn-out HCM, the thickness of the ventricular wall is reduced and the hyper-enhancement is relatively thickened, while the pattern of LGE can be similar to that of ischemic cardiomyopathy. In hypertension, LGE is related to left ventricular hypertrophy caused by pressure overload and is an independent risk factor to cardiac mortality (Rudolph et al., 2009). Moreover, myocardial fibrosis detected by LGE is associated with increased left ventricular filling pressure and longitudinal systolic dysfunction in early-stage hypertensive patients. On the other hand, hypertensive patients with increased ventricular filling pressure and diastolic dysfunction undergo LV remodeling (Contaldi et al., 2016). In cardiac sarcoidosis, LGE usually shows a non-ischemic pattern with hyper-enhancement of the mid-myocardial wall or the epicardium. However, subendocardial or transmural hyper-enhancement have also been observed, mimicking an ischemic pattern. The basal septal walls are most frequently involved, although sometimes hyper-enhancement is detected in other territories, including the RV (Komada et al., 2016). Cardiac involvement in systemic amyloidosis is frequent. On CMR, diffuse myocardial hypertrophy including both the ventricles and atria is observed with thickened valve leaflets and pericardial effusion. After gadolinium administration, there may be a circumferential pattern of LGE, preferentially affecting the subendocardium, but sometimes showing a patchier transmural pattern (Hashimura et al., 2017). For valvular heart disease, the hyper-enhancement of the myocardium may not be discoverable, since cardiac remodeling is not apparent in early progression of the disease, but it is noteworthy that valve function can be effectively evaluated by CMR. Furthermore, CMR can provide information regarding the influence of the valve lesion on the myocardium by LGE and other parameters of cardiac structure, which is very important for physicians to delineate an operation plan and evaluate the prognosis after valve replacement.

#### LGE and prognosis

LGE has provided clinicians with tools to distinguish non-ischemic from ischemic cardiomyopathies and to identify the etiology of non-ischemic cardiomyopathies. In addition, the presence of LGE and its extent in myocardial tissue relates to overall cardiovascular outcomes (Groves et al., 1989; Duan et al., 2015; Kato et al., 2015; Barbier et al., 2016; Birnie et al., 2016; Liu et al., 2016; Raina et al., 2016). Our team (Liu et al., 2016) reported that the amount of LGE from high-scale threshold on CMR correlated positively with the likelihood for adverse cardiovascular outcomes in patients with end stage C&D heart failure (adjusted hazard ratio, 1.46/10% increase in LGE; *P* = 0.002). This association was independent of the LVEF and etiology of heart failure (ischemic cardiomyopathies and non-ischemic cardiomyopathies).

Dilated cardiomyopathy (DCM) is a primary cardiomyopathy of both genetic and non-genetic origin that is characterized by dilation of the ventricle and systolic dysfunction in the absence of coronary artery disease. DCM is the most frequent indication for heart transplantation. Duan et al. (2015) performed a meta-analysis to evaluate the association between LGE and major adverse events in DCM patients. In the meta-analysis, the presence of LGE was significantly associated with all-cause mortality, cardiac death/transplantation, hospitalization for heart failure, and SCD, suggesting that LGE might provide a reference for (1) risk stratification in deciding on preventive anti-arrhythmia treatments and (2) identification of patients who would benefit from more intensive clinical care for deteriorating heart failure.

HCM is a relatively common genetic disorder of the cardiac sarcomere, characterized by an idiopathic LV hypertrophy and represents the most frequent cause of SCD among young athletes. Traditional risk factors for SCD in patients with HCM such as family history of SCD, personal history of ventricular fibrillation (VF) or ventricular tachycardia (VT), frequent non-sustained VT on 24-ambulatory holter, LV wall thickness >30 mm can sometimes be inconclusive in determining a patient's risk of SCD. Recent studies suggest that the extent of LGE (>15% of the myocardial mass) can play a major role in SCD stratification in this subset of patients (Chan et al., 2014). A recent meta-analysis (Briasoulis et al., 2015) that included over three thousand individuals (*n* = 3067) with HCM, highlighted the relationship between LGE and cardiovascular mortality in HCM. This study further proves that extensive LGE is a promising risk-stratification tool as it significantly predicts SCD risk, cardiac mortality, and all-cause mortality in patients without conventional risk markers. Hence, LGE should be considered a novel risk marker in predicting SCD in HCM. Currently available data support that patients with extensive LGE for primary prevention of SCD should be considered for ICD implantation, even if they are not at high-risk according to the conventional risk markers.

Sarcoidosis is a systemic multi-organ disorder of unknown etiology that is histopathologically characterized by granulomas. Clinical cardiac involvement is detected in only 5–7% of patients with sarcoidosis, whereas postmortem studies have identified myocardial lesions in 20–60% (Satoh et al., 2014). In sarcoidosis, patients with LGE in the myocardium show heart failure symptoms and a higher prevalence of ECG abnormalities and VTs. However, several recent CMR studies suggested the presence and extent of myocardial LGE as an even more important overall prognostic factor than LV function (Birnie et al., 2016).

Cardiac amyloidosis is a common cause of restrictive cardiomyopathy, and reduced ventricular wall compliance leads to impairment of diastolic filling and diastolic heart failure even when systolic function was preserved. A recent systematic review and meta-analysis supported that the incremental prognostic value of LGE. Thus, LGE may be useful for risk stratification of these patients for aggressive medical management, possibly leading to a decrease in all-cause mortality (Raina et al., 2016).

Despite the aforementioned uses, currently, no “best” threshold can perfectly coincide with the extent of fibrosis because the presence of LGE depends on many different thresholds. Additionally, the LGE signal intensity is not precise enough to differentiate the types of fibrosis (i.e., interstitial fibrosis and replacement fibrosis). Moravsky et al. explored the association between the extent of LGE at different thresholds and the degree and content of fibrosis in myocardial tissues of patients with HCM. LGE quantified by 5 *SD* has a better association with interstitial than with replacement fibrosis, whereby the LGE measured by 10 *SD* may show a stronger correlation with replacement fibrosis. However, the authors underscore that LGE quantified by a high threshold cannot reflect the type of fibrosis because it represents the sum of the two types (Moravsky et al., 2013). Current T1 mapping techniques, including quantification of extracellular volume (ECV), can be used to overcome the drawback of LGE imaging in the set of diffuse myocardial diseases.

#### T1 mapping

T1 mapping has prominent advantages to detect diffuse fibrosis, since it assesses the T1 relaxation time of myocardial tissue. Several studies showed that the association of T1 mapping (including native T1, post-contrast T1, and extracellular volume fraction) with CVF in myocardial biopsies of AS (Everett et al., 2016; Taylor et al., 2016). Kockova et al. showed that native T1 relaxation time is related to diffuse myocardial fibrosis of severe heart valve disease, primarily AS, since the native T1 was prominently higher than in the control group (Kockova et al., 2016). Ellims et al. found that post-contrast T1 measurements in cardiac transplant recipients associated with invasively demonstrated LV stiffness, which was likely to be a major contributor to diastolic dysfunction (Ellims et al., 2014).

Extracellular volume fraction (ECV), a novel parameter evaluating myocardial fibrosis, is measured from both pre- and post-contrast T1 mapping analyses. In a recent study, Barison et al. found that the myocardial ECV in patients with non-ischemic dilated cardiomyopathy was higher than that in normal individuals, and the extent of ECV was associated with worsening left ventricular function and composite events such as VT. More importantly, ECV could identify early interstitial fibrosis with no apparent LGE, which provides physicians with a valuable way to predict the prognosis of HF patients with non-ischemic dilated cardiomyopathy (Barison et al., 2015). ECV could replace LGE-CMR to detect diffuse and microscopic myocardial fibrosis, which is barely visualized in LGE-CMR. aus dem Siepen et al. showed that ECV reflects the level of myocardial fibrosis in patients with DCM, and the ECV in patients with earlier-DCM without LV dysfunction is significantly changed (aus dem Siepen et al., 2015). ECV may be an important method to assess mechanistic and hemodynamic abnormalities in patients with HCM. van Ooij et al. demonstrated that altered left ventricular out tract hemodynamics associated with ECV in patients with HCM. Therefore, LV remodeling may contribute to LV flow abnormalities and increasing LV loading (van Ooij et al., 2016). Moreover, ECV is increased in HCM sarcomere mutation carriers, even before left ventricular hypertrophy becomes detectable. Myocardial fibrosis is a trigger factor for HCM and early detection of ECV in sarcomere mutation carriers can help diagnosing HCM. Based on the above two studies, targeting fibrosis could be a promising therapy for HCM (Ho et al., 2013). Presently, there are only few studies on ECV, but the future of this technique is promising and more studies are being devoted to it.

Despite these advantages, the limitations of CMR need to be considered. For patients who have pacemakers or defibrillators, CMR cannot be applied to evaluate the myocardium lesion. In addition, because LGE-CMR needs gadolinium-based contrast agents to show the matrix, the risk of worsening renal dysfunction for patients with renal insufficiency should be considered by clinicians to avoid further renal injuries (Karamitsos et al., 2009). However, CMR is still the best method to evaluate myocardial fibrosis, although more techniques are evolving to differentiate the types and extent of myocardial fibrosis. Thus, clinical trials need to select more precise techniques that are consistent with practical fibrosis and further demonstrate the association between the markers of fibrosis and its prognosis.

### Histology and CVF

Although CMR can effectively detect and evaluate the degree of myocardial fibrosis, histology of endomyocardial biopsy specimens from patients with HF is still considered the gold standard for myocardial fibrosis detection. In some studies, the CVF is calculated to evaluate the extent of myocardial interstitial fibrosis. The CVF is the ratio of the sum of all connective tissue areas over the sum of all connective tissue and muscle areas from averaged values of several representative fields of the tissue section. CVF can qualitatively determine the extent of myocardial fibrosis, but the cut-off values of mild and severe fibrosis have not been defined, and the cut-off varies from study to study. Aoki et al.'s study proved that, after excluding ischemic and valvular etiologies, the CVF obtained from LV endomyocardial biopsy samples had a prognostic impact in HF patients with HFrEF rather than for those with HFpEF (Aoki et al., 2011). The biggest disadvantage of an endomyocardial biopsy is that it may not detect regional myocardial fibrosis, so sampling in different regions may be essential to detect patchy myocardial fibrosis. However, according to moral and ethical regulations, these methods may not be effectively applied or generalized.

## Treatments

During the past several decades, the medical treatment of HF has dramatically progressed. Traditional medical interventions to improve the prognosis of HF are beta-blockers, ACE inhibitors, and aldosterone antagonists. These three first-line interventions are called the “Golden Triangle” of HF treatment. Beta-blockers upregulate the expression of beta-adrenergic receptors (β-AR) and increase the sensitivity of the ligand to the β-AR. Moreover, beta-blockers have certain antiarrhythmic effects, which decrease the risk of SCD. ACE inhibitors target the renin-angiotensin-aldosterone system and inhibit cardiac remodeling. Losartan, an AT_1_receptor antagonist, can suppress the function of TGF-β1 to prevent activation of myofibroblast and the fibrosis process through blocking Ang II binding to the AT_1_R on myofibroblast (Sun et al., 1998). Although almost all AT_1_ receptor blockers improve the prognosis of patients with HF, the mortality and morbidity rates of HF remain high, which motivates researchers to comprehensively explore the pathological processes of HF and new target sites for treatment. Myocardial fibrosis, a neglected target site, provides a novel direction for HF treatment.

### New pharmaceutical target sites

#### Drugs targeting collagen synthesis and cross-linking

Serelaxin, a recombinant form of human relaxin-2, can inhibit the activation of fibroblasts and decrease collagen production to suppress the fibrotic process (Table 2; Tietjens and Teerlink, 2016). An *in vivo* animal experiment discovered that serelaxin could effectively suppress myocardial fibrosis better than the ACE inhibitor, enalapril. Serelaxin has entered phase III clinical trials for HF treatment and has been found to improve dyspnea and the 180-day mortality rate in patients with acute HF (Samuel et al., 2014).

**Table 2:** **New interventions and their targets of myocardial fibtosis**.

**New interventions**	**Targets**	**Mechanisms**
**DRUG**		
Serelaxin	Relaxin 2	Inhibit the activation of fibroblasts and collagen collagen production
AntimiR-21	miRNA-21	Reduces interstitial fibrosis and attenuates cardiac dysfunction
AntimiR-208a	miR-208a	Suppress fibrosis
MMPs inhibitor	MMPs	Inhibit pathological cardiac remodeling
**MECHANOTHERAPY**		
CRT		Reverse remodeling

On the other hand, suppressing pre-collagen from maturing and crossing-linking are also promising directions for HF treatment. Some studies indicated that the loop diuretic, torasemide, may inhibit lysyl oxidase to limit the speed at which collagen crosslinks. Therefore, more studies are needed to identify the role of this drug in reducing myocardial fibrosis. Another study showed that torasemide can reduce myocardial fibrosis in patients with chronic HF and can decrease PICP activation of the TGF-β1 signaling pathway (Lopez et al., 2007). An anti-TGF-β1 antibody can also suppress this signaling pathway to decrease collagen synthesis (Wynn and Ramalingam, 2012), but this method is not specific to the heart; thus, inhibiting the TGF-β1 signaling pathway systemically may bring unexpected outcomes in attempting to suppress myocardial fibrosis.

Moving to miRNAs has become popular in recent years, including miRNA mimicry and anti-miRs (Table 2). Though knocking down related genes can also inhibit targeted miRNAs, researchers advocate for anti-miRs, which are several oligonucleotides that bind to miRNAs. Different transforming forms of anti-miRs can increase cellular uptake and the affinity of related miRNAs (van Rooij and Olson, 2012). For example, the miR-21 antagonist reduces interstitial fibrosis and attenuates cardiac dysfunction induced by pressure overload in mice (Thum et al., 2008). Montgomery et al. found that a LNA-oligonucleotide inhibiting the cardiac miR-208a specifically could suppress fibrosis and improve survival in rats on a high-salt diet (Montgomery et al., 2011). However, targeting miRNAs is still at the stage of fundamental research and still need to be studied further. Moreover, targeting miRNAs can upregulate the expression of various downstream mRNAs whose functions may not be presently known and may be the source of their side effects, thereby limiting their development by pharmaceutical companies.

#### Drugs targeting collagen degradation

MMP inhibitors, including non-selective and selective inhibitors, have been used to pharmacologically treat HF (Table 2). For example, batimastat, marimastat, GM-6001 (ilomastat or gelardin), PD-166793, and ONO-4817 are non-selective inhibitors of MMPs. Some studies found that selective inhibitors of MMP-9 and MMP-2 are non-stable in the body due to proteolysis. However, semi-selective inhibitors (PY-2 and 1, and 2-HOPO-2) produce better results than the non-selective inhibitors, PD166793 and CGS27023A, in improving ischemia reperfusion injury. Chemically modified tetracycline-3 (CMT3) is devoid of its antimicrobial properties, but can inhibit MMP-2 and MMP-9, which inhibit pathological cardiac remodeling (Mishra et al., 2013). More studies are needed to understand the pathophysiology of this approach to identify more effective target sites.

### Mechanotherapy

Cardiac resynchronization therapy (CRT) and implantable cardioverter defibrillators have been applied for the treatment of HF. HF can be prevented in part by limiting the occurrence of malignant arrhythmias either directly by using implantable cardioverter defibrillators, or by improving the coordination and contractility of cardiac muscles by CRT. Both interventions can further improve the prognosis of HF with fundamental medical interventions. In 2013, the ACCF and other cardiac organizations issued the Guidelines of Appropriate Use Criteria for Implantable Cardioverter Defibrillators and Cardiac Resynchronization Therapy, which provides clinicians the ability to decide therapeutic schedules in different situations. Some studies showed that CRT can produce reserved remodeling, which is defined as a reduction in LV end-systolic volume at 6 months after CRT (Table 2; Russo et al., 2013). Although there are still not enough studies to evaluate the impact of CRT on myocardial fibrosis, Bose et al. and Ypenburg et al. reported that the extent of scar tissue and viable myocardium were directly related to the response of CRT (Ypenburg et al., 2007; Bose et al., 2014). Furthermore, Leong et al. reported that the extent of myocardial fibrosis is independently associated with the lack of response to medical therapies in a new presentation of DCM (Leong et al., 2012).

## Conclusion

Despite the significant amount of research now addressing myocardial fibrosis, the understanding of its pathogenesis, clinical implications, and its management remain limited. In terms of the pathophysiology of myocardial fibrosis, more governing factors should be further explored to improve diagnostic methods and identify therapeutic targets. Nonetheless, myocardial fibrosis carries clinical prognostic importance. Serum markers can be used to significantly assess myocardial fibrosis, but whether they should be applied in a clinical setting is still a matter of debate. LGE is an established tool to assess the extent of myocardial fibrosis, but there is no specific cut-off value to evaluate its prognosis. Additionally, ECV represents a novel technique that has promising future for diagnosing myocardial fibrosis. CVF, the golden standard for myocardial fibrosis detection, cannot be easily generalized because of its invasive nature. Currently, there is no specific medical targeted therapy to reduce myocardial fibrosis. However, more clinical experiments have been devoted to the known target sites of myocardial fibrosis and the development of myocardial pathogenesis for the discovery of new medical interventions. Future studies should focus on the understanding of the pathophysiological mechanisms underlying myocardial fibrosis, identify targets of potential therapy, and improve intervention strategies.

## Author contributions

TL and DS select articles about the pathogenesis of mycardial fibrosis, and wrote the review. PZ, JD, and TL were responsible for guiding clinical manifestation and treatment of heart failure. XM and WL contributed to recommend high-quality articles about late gadolinium enhancement and helped to accomplish the write about cardiac magnetic resonance imaging. SL and JL guided writing of the pathogenesis. LZ revised the clinical part of manuscript.

## Funding

This review was supported by the China Natural Science Funding (81101173 and 81470429) and the High Levels of Health Technical Personnel in Beijing City Health System (2013-3-005).

### Conflict of interest statement

The authors declare that the research was conducted in the absence of any commercial or financial relationships that could be construed as a potential conflict of interest.
